# Protective Effects of a Polyphenolic Phytochemical Quercetin against Oxidative Dysfunctions in Rats

**DOI:** 10.1155/2023/7858718

**Published:** 2023-04-20

**Authors:** Ahmed I. Foudah, Mohammad A. Salkini, Hasan Soliman Yusufoglu, Huda Mohammed Alkreathy, Rahmat Ali Khan

**Affiliations:** ^1^Department of Pharmacognosy, College of Pharmacy, Prince Sattam Bin Abdulaziz University, Al-Kharj, Saudi Arabia; ^2^Department of Pharmacognosy & Pharmaceutical Chemistry, College of Dentistry & Pharmacy, Buraydah Private Colleges, Buraydah 51418, Saudi Arabia; ^3^Department of Pharmacology, Faculty of Medicine, King Abdulaziz University, Jeddah, Saudi Arabia; ^4^Department of Biotechnology, University of Science and Technology Bannu, Bannu, KPK, Pakistan

## Abstract

**Background:**

Quercetin hastraditionally been used in various oxidative and urinary tract dysfunctions. Thecurrent project is consequently set to evaluate the defensive efficacy ofQuercetin against potassium bromate (KBrO3) induced testiculartissue oxidative dysfunctions through biochemical, hormonal, and genotoxicmarkers.

**Methods:**

To observe theprotective efficacy of Quercetin against urinogenital oxidative dysfunction inrats, thirty six albino male rats were divided into six groups. Protectiveefficacies of Quercetin were checked on reproductive hormonal levels,antioxidant enzyme activities, lipids peroxidation (LP), and DNA damages.

**Results:**

Potassium bromate exposure in experimentalanimals caused a reduction in the activities of antioxidant enzymes and disturbedhormonal secretions while enhancing the peroxidation of lipids andfragmentations of DNA. Cotreatment of Quercetin considerably (P<0.01)reversed these abnormalities with admiration to levels of hormones, antioxidantenzymes activities, and peroxidations of lipids secure to those seen inuntreated rats. (*P* < 0.01)

**Conclusion:**

The findings of the current project revealedthat various doses of Quercetin are able to keep the testicular organ fromabnormal free radical dysfunctions. These improvements might be due to theantioxidant ability of polyphenolic bioactive constituent, i.e., Quercetin.

## 1. Introduction

Oxidative stress and dysfunction inside the cell take place as soon as the meditation of reactive oxygen species productionexceeds the system's antioxidant capability. In the aging process, oxidativestress plays an important role, and many pathogeneses are responsible fordiseases such as diabetes, cancer, neurodegenerative diseases, and respiratorydiseases [1]. _3_Various toxic reports revealed that KBrO3an oxidizing agent causes hepatotoxicity, and mesothelioma tumor development ininvestigational animals causes thyroid, kidney failure, andneurotoxicity [2]. _3_Various toxic reports revealed that KBrO3, an oxidizing agent, causes hepatotoxicity and mesothelioma tumor development ininvestigational animals, as well as causes thyroid, kidney failure, andneurotoxicity [3]. Experimental models have been the subject ofnumerous studies on oxidative injury and KBrO3-investigatingpossible mechanisms of induced carcinogenicity _3_ [4]. The KBrO3 produced reactive speciescombined with polyunsaturated fatty acids (PUFA) present in the tissue membraneto form DNA fragments [5]and decrease the activities of antioxidantenzymes and nonenzymatic antioxidants [6]. To prevent pathology, it is necessary tosupply external antioxidant compounds and maintain a balance between oxidantsand antioxidants. However, conventional and synthetic drugs used to treat oxidativestress are sometimes inadequate and can have many side effects [7]. However, most consumers prefer to use natural,more effective antioxidants for a safer approach. Accordingly, plant extractsand their metabolites such as flavonoids, terpenoids, and phenolic components,provide an opportunity in this regard [8]. The use of natural antioxidants to combat tissuedamage has been suggested as a healing agent as well as a coagent of medicine.Quercetin is used in the treatment of different types of cancer [9, 10], inflammation [11], oxidative damage [12], and antitumor effects [13]. Therefore, we designed to explore the protectingrole of Quercetin against potassium-bromated induced testicular carcinogenesisin rats.

## 2. Materials and Methods

### 2.1. Experimental Method

The present project is composed of thirty albinomale rats were divided into 06 groups, each group containing 06 rats:

  Group I as a control group.
  Group II wasgiven a 3 ml/kg DMSO dose.
  Group III wasgiven high grade 20 mg/kg KBrO3._3_
  Group IV wascoadministered 75 mg/kg quercetin after 48 hrs of KBrO3 treatment._3_
  Group V wascotreated with 150 mg/kg bw quercetin after 48 hrs of KBrO3treatment._3_
  Group VI wasgiven 150 mg/kg quercetin alone.

_2_For four weekstreatments were given twice a week. Upon the end of the experiment, all animalswere kept on a normal diet for 24 hrs without any treatment. The animals wereanesthetized, and blood was isolated from the ventral side and collected into afalcon tube, centrifuged, and refrigerated. Then the testicular tissue was removed anddried with blotting papers and weighed. After tissue coagulation, it wasdivided into 2 parts. For histology, one portion was cut and frozen anotherpart at -70°C after treatment with liquid N2 for further molecular andbiochemical studies.

### 2.2. Serum Biochemistry

Variousparameters of serum including endocrine hormones such as testosterone,estradiol, luteinizing hormones (LH), and follicle-stimulating hormones (FSH)and prolactin were calculated using a kit purchased from 10227-Czech Republic(IM1447-IM3286) IMMUNOTECH Company for serum levels.

### 2.3. Antioxidant Profile

 (*γ*80 mg of tissue was homogenized in phosphate bufferat 4ºC and centrifuged at 10,000 rpm. Then, from the upper clear phase, totaltissue protein, glutathione (GSH), thiobarbituric acid reactive substances(TBARS) levels, catalase (CAT), peroxidase (POD), superoxide dismutase (SOD),glutathione peroxidase (GSH-Px), gamma glutamyl transferase (γ-GT), glutathionereductase (GSR), and glutathione-S-transferase (GST) activities were measuredas described [14–23].

### 2.4. Genotoxicity Assays

Quantitative DNA damages were estimated usingthe protocol of Lee and Jeong [24].

### 2.5. Histopathological Studies

Cellular changeswere observed under a light microscope at 40x.

### 2.6. Statistical Analysis

To determinetreatment effects, the variables were analyzed unilaterally using SPSS13.0, a well-known computer software. Thesignificance levels in different treatments were determined by LSD at 0.05% and0.01% probability levels.

## 3. Results

### 3.1. Effects of Quercetin on Reproductive Hormonal Secretions

**Figure 1:**
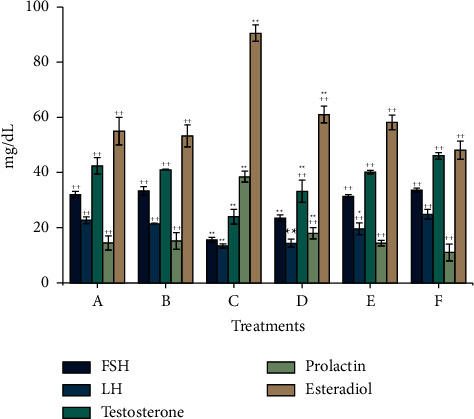
The effect of various doses of quercetin on serum male hormones in rat.

Hypothalamuses, the pituitary axis (HPA axis) ofhormonal secretion were highly affected by ROS. The effect of different dosesof Quercetin on serum levels of endocrine hormones such as testosterone,estradiol, luteinizing hormones (LH), and follicle-stimulating hormones (FSH)and prolactin were shown in Figure 1. Potassium bromated increased (*P* < 0.01)hormonal secretions of FSH, prolactin, andestradiol comparatively normal control group. Subsequent treatment withdifferent doses of Quercetin significantly eliminated the toxic effects of(*P* < 0.01) KBrO_3_KBrO3 and improved near-control serumlevels of prolactin and estradiol. Serum levels of FSH, testosterone, and LHwere significantly increased (*P* < 0.05, *P* > 0.01) at 75 mg/kg and 150 mg/kg by treatment ofQuercetin. Administration ofQuercetinwas more potential as it significantly restored the serum levels of luteinizinghormones and follicle stimulating hormones with 75 mg/kg and 150 mg/kgtreatment in the control group (*P* < 0.01).

### 3.2. Effects of Quercetin on Tissue Homogenate Protein, SOD, POD, and CAT Activity

**Figure 2:**
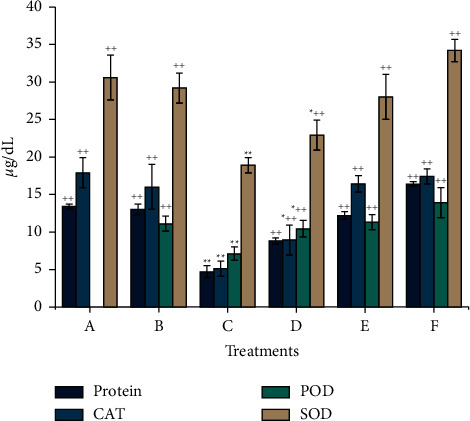
The effect of various doses of quercetin on testis protein, CAT, POD, and SOD.

Administrations of Quercetin in different doseson tissue proteins and antioxidant enzymes such as POD, SOD, and CAT effects wereshown in Figure 2. The concentrations of soluble tissue protein andthe activity of SOD, POD, and CAT were significantly reduced by the treatment ofpotassium bromated. Coadministration of various doses of Quercetin recoveredthese abnormalities and maintained (*P* < 0.01) near the control group (*P* < 0.01).

### 3.3. Effects of Quercetin on QR, *γ*-GT, GSH-Px, GST, and GSR Activity

**Figure 3:**
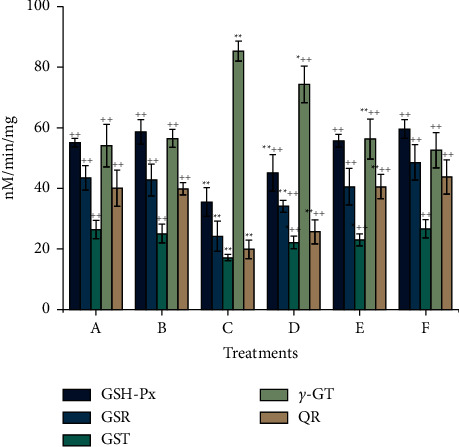
The effect of various doses of quercetin on testis GST, GSR, GSH-Px, *γ*-GT, and QR.

The protective effects of different doses of quercetin against KBrO_3_ at different enzyme activities such as QR, *γ*-GT, GSH-PX, GST, and GSR are shown in Figure 3. In rats, 20 mg/kg BW of KBrO_3_ significantly (*P* < 0.01) reduces the activity of phase-II metabolic enzymes such as GST, GSR, and GSH-Px and increases (*P* < 0.01) the activities of *γ*-GT and QR. After treatment with different doses of quercetin, enzyme activity was significantly restored near the control group (*P* < 0.01).

### 3.4. Effects of Different Doses of Quercetin on GSH, TBARS, H_2_O_2_, and Nitrate Content

**Figure 4:**
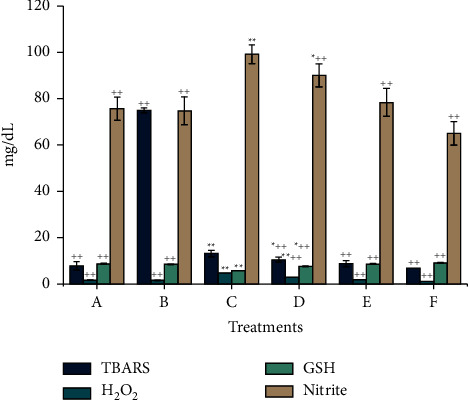
The effect of various doses of quercetin on testis GSH, TBARS, H_2_O_2_, and nitrite contents.

Figure 4 presented the content of GSH, TBARS, H_2_O_2,_ and nitrates in tests of different experimentalgroups of rats. Lipid peroxidation is caused by 20 mg/kg KBrO_3_ and significantly reduces GSH content (*P* < 0.01) while increasing nitrate, TBARS, and H_2_O_2_ content as compared to the control group. The content of GSH, tissue nitrate, TBARS, and H_2_O_2_ was significantly higher (*P* < 0.01) than in the control group. Cotreatment of various doses of quercetin caused all these contents to improve to a normal level.

### 3.5. Effects of Quercetin on Testis Weight, Relative Weight, and % DNA Fragments

**Figure 5:**
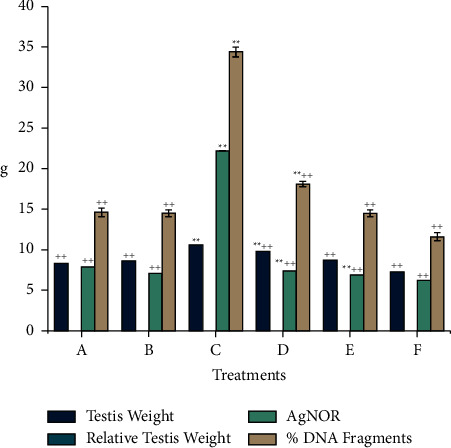
The effect of various doses of quercetin on testis weight, relative testis weight, AgNORs, and % DNA fragmentation.

The effects of Quercetin against KBrO_3_ toxicity on testis weight, relative testisweight, AgNORs, and DNA damages were shown in Figure 5. Administrations of KBrO_3_ caused abnormalities in tissue weight, relative tissue weight, % DNA damage, and AgNORs. Cotreatment of rats with different doses of quercetin significantly improved (*P* < 0.01)tissue and relative tissue weight, DNAfragments, and number of NORS per cell near the control group.

### 3.6. Effect of Quercetin on the Histopathology

Microscopic examinations of the malereproductive system of the control group showed normal shape seminiferoustubules and sperm concentrations. Sertoli cells were not clear. Stromaappearance and histological appearance of fibrous muscle surrounding theprostate gland were found to be normal. Administration of KBrO_3_ caused degeneration ofsemeniferous tubules, aberration of epithelium, obstruction of meiosis, andabnormal shape and concentration of semen. Administration of different doses ofQuercetin revealed clear repair of testicular abnormalities induced by KBrO_3_ near the control group as shown in Table 1.

## 4. Discussions

Medicinal plants play an important role in thedetoxification of free radicals due to the presence of bioactive ingredients.In the present study, it was reported that various doses of Quercetin havesignificantly reversed the KBrO_3_-induced pancreatic stress. Induction of KBrO_3_ generated free radicals that caused the productionof highly reactive trichloroethylene and peroxy radical by the system ofcytochrome P_450_ oxygenase trigger the initiation of lipidperoxidation [24]. Lipid peroxidation further causes strandbreakage and DNA mutation [24]. In the present study, KBrO_3_ caused DNA damages in the testis, which weresignificantly improved by various doses of Quercetin. Related to thesefindings, other reports revealed that plant extract and its various componentscomprehensively improved injuries caused by KBrO_3_ intoxication and DNA strand breakage [1].

Free radicals are thought to cause cellularinjuries through lipid peroxidation [25]. In the current study, coadministration ofvarious doses of Quercetin considerably reversed the serum markers such asinsulin, lipase, and amylase as well as blood glucose levels. Our results werein agreement with other findings [26], which reported that the same effect may be due to antioxidant activity.

SOD, POD, and CAT are highly effectiveantioxidant enzymes responsible for the catalytic distribution of highlyreactive toxic radicals viz; superoxide and peroxide radicals [27–29]. In the present study, induction of KBrO_3_ caused depletion of this enzymatic level whichwas significantly modulated by various doses of Quercetin. The glutathionesystem includes GSH, GSH-px and GSH, hydrogen peroxide, and hydroperoxide causesdeficiency [30–32]. In the present study, various doses of Quercetinsignificantly reversed the reduction in the enzymatic level of GST, GR, GPx, andquinone reductase, which were depleted by induction of KBrO_3_. Similar observations were reported during theadministration of the chemical stimulant *Coriandrum sativum,* against oxidative stress [33, 34].

Lipid peroxidation (LPO) is an automated processand can result in peroxidative tissue damage inflammation, cancer, and aging asa common result of cell death [35, 36]. In the present study, administration of KBRO_3_resulted in a significant increase in tissue MDAconcentration indicating lipid peroxidation. Interestingly, the combinedadministration of different doses of Quercetin has significantly reduced theLPO threshold by significantly reducing the MDA concentration, which is theeffect of the extract against the lipid peroxidation of the tissue induced byKBrO_3_. Similar reports have been documented in various studies [37, 38].

The administration of KBrO_3_ revealed abnormal cellular changes in testiculartissue. Coadministration of different doses of Quercetin showed protectiveeffects and reduced cell degeneration. Our study revealed a similarinvestigation that agrees with previous findings [39] while examining the protective effect of medicinal plants against KBrO_3_-induced toxicity in rats.

## 5. Conclusion

The finding of the current study showed thatvarious doses of Quercetin are strong antioxidant and is capable to protecttesticular damage from KBrO_3_-induced toxicity. However, further studies areneeded on the subject to study the mechanism of action.

## Figures and Tables

**Table 1:** Effect of various doses of quercetin on histopathology.

Treatment	Tubules blockage	Meiotic interruption	Somniferous tubules	Germinative epithelium
Control	—	—	—	—
DMSO	—	—	—	—
20 mg/kg KBrO_3_	++	++	++	++
75 mg/kg quercetin + KBrO_3_	−/+	—	—	−/+
150 mg/kg quercetin + KBrO_3_	—	—	−/+	—
150 mg/kg quercetin alone	—	—	—	—

—, normal; −/+, mild; ++, medium.

## Data Availability

All the data and material relevant to the paper are available in the paper.
